# Medical Diplomas

**Published:** 1881-05

**Authors:** 


The following bill has recently passed the Ohio Legislature and is now a law:

Section 1. *Be it enacted by the General Assembly of the State of Ohio,* That the following sections be enacted as supplementary to Section 4403, of the revised statutes, with sectional numbering as herein provided:

Section 4403*a*. Whoever shall make, issue, or publish, for purpose of sale, barter or gift, any certificate, diploma, or other writing, or document falsely representing the holder or receiver thereof to be a graduate of any medical school, or college, or of any educational institution of medicine whatsoever, and entitled to the powers, privileges, or degrees thereby pretended to be conferred; or whoever shall sell, or otherwise dispose of, or offer to do so, any such diploma, certificate, writing, or document containing the false representation aforesaid; or whoever shall use his name or permit the same to be used, as a subscriber, for any purpose or in any capacity, to such false and fictitious diploma, certificate, writing, or document aforesaid or whoever shall engage in the practice of medicine and surgery under and by virtue of such fraudulent diploma, certificate, writing, or document aforesaid, shall be deemed guilty of a misdemeanor, and, upon conviction thereof, shall be subject to the penalty prescribed in Section 4403*b*.

SECTION 4403*b*. Whoever shall make, issue, or publish, or cause to be made, issued, or published, for the purpose of sale, barter, or gift, any diploma, certificate, or writting representing the holder thereof to be a graduate of any medical school, or college, or of any educational institution of medicine whatsoever, unless such holder shall have, in fact, attended a complete course of instruction in such school, college, or institution for medical teaching, equal to the average course of instruction in other schools, colleges, or institutions, where the various branches of medicine is taught as a science, in good standing in the state of Ohio, shall be deemed guilty of a misdemeanor, and, upon conviction thereof, shall be fined in any sum not exceeding one thousand dollars, nor less than one hundred dollars, or imprisoned in the penitentiary not more than three years, nor less than one year, or both, at the discretion of the court.

SECTION 2. This act shall take effect and be in force from and after its passage.

We hope the present legislature will continue the good work by enacting a registry law and a law establishing a State Board of Health.—*Cincinnati Lancet and Clinic.*

